# Efficacy of split-thickness skin graft combined with novel sheet-type reprocessed micronized acellular dermal matrix

**DOI:** 10.1186/s12893-022-01801-x

**Published:** 2022-10-11

**Authors:** Hyung Min Hahn, Yon Soo Jeong, Il Jae Lee, Min Ji Kim, Hyoseob Lim

**Affiliations:** Department of Plastic and Reconstructive Surgery, Ajou University School of Medicine, 164, World cup-ro, Yeongtong-gu, Suwon, 16499 Republic of Korea

**Keywords:** Split-thickness skin graft, Acellular dermal matrix, Graft contracture, Dermal substitute, CGDerm Matrix, Reprocessed micronized ADM

## Abstract

**Background:**

Autologous split-thickness skin grafts (STSGs) remain the mainstay for treatment of large skin defects. Despite its many advantages, there exist critical disadvantages such as unfavorable scar and graft contracture. In addition, it cannot be used when structures such as tendons and bones are exposed. To overcome these limitations, acellular dermal matrix (ADM) is widely used with STSG. CGDerm Matrix^®^, which was recently developed, is a novel reprocessed micronized ADM (RMADM). In this study, outcomes of the combined application of RMADM and STSG on full-thickness wounds were analyzed.

**Methods:**

Forty-one patients with full-thickness skin defects due to trauma, scar contracture release, and diabetic foot ulcers, who underwent STSGs, from January 2021 to July 2021, were retrospectively reviewed. The primary outcome of interest was skin loss rate, which was measured 14 days after surgery.

**Results:**

The most common cause of skin defect was trauma (36 patients), diabetic foot (2 patients), scar contracture release (2 patients), and malignancy (1 patient). The average defect size was 109.6 cm^2^ (range, 8–450 cm^2^). The average skin loss rate was 9.1%, showing a graft take rate of > 90%.

**Conclusion:**

The use of combined RMADM and STSG in full-thickness wound reconstruction provides stable and acceptable outcomes. The newly developed ADM can be a promising option in wound reconstruction.

**Supplementary Information:**

The online version contains supplementary material available at 10.1186/s12893-022-01801-x.

## Background

In case of full-thickness skin defects, plastic surgeons make decisions according to the reconstructive ladder. Split thickness skin graft (STSG) is located on the lower rung of the reconstructive ladder and can be used to reconstruct large skin defects. Therefore, autologous STSGs are still considered the mainstay for the treatment of large skin defects due to trauma and scar contracture release [1].

Despite the many advantages of STSG, fatal disadvantages exist. The grafted skin may not take well to the surrounding tissues, and there may be limitation of motion if the wound is close to the joints due to graft contracture. In addition, the texture of the grafted skin is different from that of normal skin, and an unfavorable scar such as a hypopigmented or hyperpigmented scar may sometimes remain [2].

To overcome these limitations, flap surgery and full-thickness skin grafts may be used; however, these techniques cannot be used as widely as STSG. A dermal substitute or acellular dermal matrix (ADM) is another frequently method to overcome the limitations of STSG [2–6].

ADM is developed by removal of the dermal cells and leaving the extracellular matrix (ECM) and basement membrane components. As ADM contains collagen and elastin, and no cells, transplant rejection does not occur [7].

However, in case of single-stage surgery with simultaneous ADM grafting and STSG, plasma imbibition interference caused by ADM can increase graft loss and the period until graft stabilization. In particular, this phenomenon occurs more easily if the mesher is not used.

**Fig. 1:**
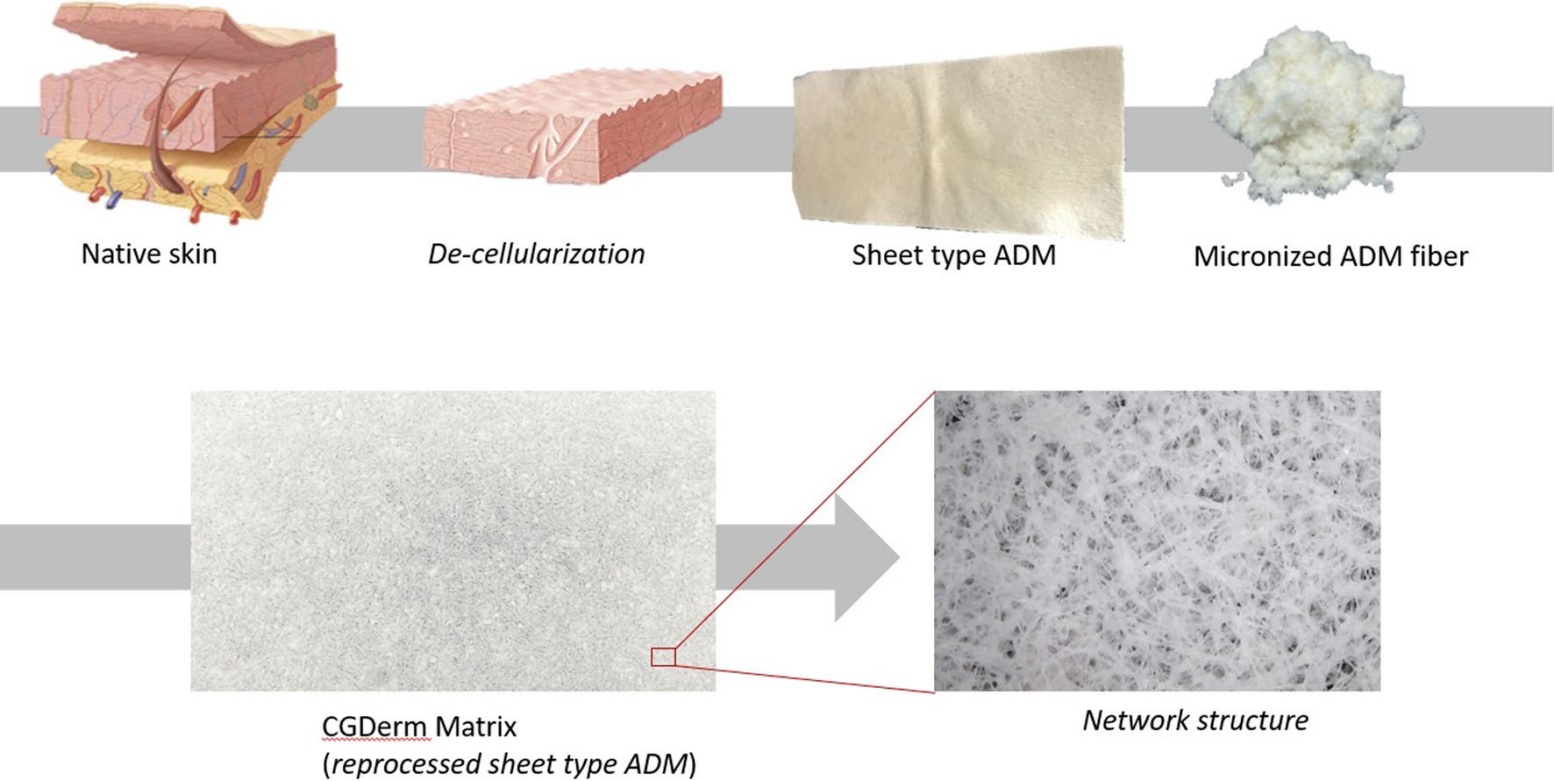
The reprocessed micronized acellular dermal matrix. Based on native skin, micronized ADM fiber was acquired after de-cellularization. Reprocessing with freeze-drying method, RMADM with network structure was made

**Fig. 2:**
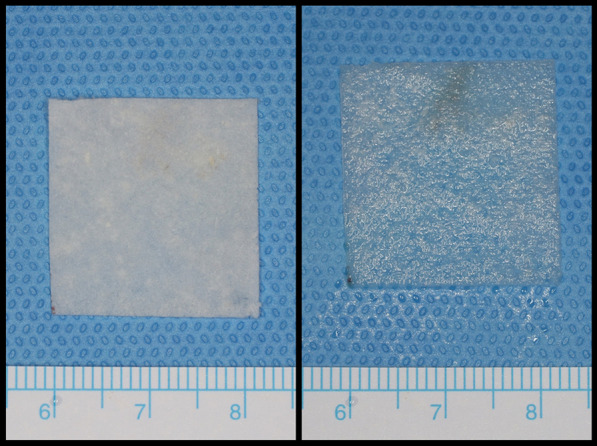
RMADM with ruler. (Left) Original status. (Right) Hydrated with saline

CGDerm matrix® (CGDM) (CGBio Co., Dae-woong Pharm., Seoul, Korea) is a reprocessed micronized ADM (RMADM) and is expected to overcome the disadvantages of the existing ADM by freeze-drying, pulverizing, and reprocessing the ADM in the form of a sheet (Figs. 1, 2). This study attempted to report the results and complications of patients who underwent STSG using RMADM.

## Methods

### Patients and protocols

From January 2021 to July 2021, patients with full-thickness skin defects due to trauma, scar contracture release, and diabetic foot ulcers were included in the study. Exclusion criteria were incomplete data, severe uncontrolled diabetes mellitus, peripheral vascular disease, and co-existing osteomyelitis. The study was conducted after obtaining approval from the institutional review board (IRB) of Ajou University Hospital.(AJIRB-DEV-INT-21-138) and the research was conducted according to the World Medical Association Declaration of Helsinki.

### Surgical protocol

STSGs were performed by four plastic surgeons. The STSG was conducted with the same surgical protocol and all the surgeons are skilled plastic surgeons registered with the board of Korean Society of Plastic and Reconstructive Surgeons. Traumatic wounds or diabetic foot ulcer wounds underwent several rounds of debridement before surgery and necrotic tissue was removed under sterile conditions. Thereafter, the growth of granulation tissue was promoted through negative pressure wound therapy (NPWT). According to the results of deep tissue culture before surgery, patients received appropriate antibiotics before surgery, and for surgeries such as scar contracture release, 1 g of cefazolin was administered intravenously within 2 h before surgery. Antibiotics were maintained for approximately 10–14 days after surgery. The timing of STSG was decided when the wound bed was clean and covered with well-vascularized tissues. All patients underwent RMADM and autologous STSG, whereas some underwent two-stage surgery. Autologous STSG (8–12/1000 of an inch in thickness) was harvested from the thigh using an electric dermatome (Zimmer Air Dermatome; Zimmer Inc., Warsaw, Poland). Unmeshed, 1:1, or 1:2 meshed STSGs were applied at the surgeon’s discretion. The RMADM was attached to the wound without hydration, and saline irrigation was performed for the RMADM. STSG was applied after the saline irrigation, and a skin stapler or #4 − 0 nylon was mainly used for skin fixation. NPWT with 120 mmHg of continuous pressure was selected as the dressing method for most cases, except in some wherein tie-over dressing was selected due to surgical site bleeding. When using NPWT, Easytact^®^ (CGBio Co., Dae-woong Pharm.), a multipore silicone wound contact layer, was used. Immobilization was performed using a splint depending on the surgical site. All medical photos were taken using the same camera, lens, and ring flash (Canon 750D camera, EF 50 mm F2.5 Compact Macro lens, MR-14EX II flash) in manual mode with the same settings. In order to minimize the change caused by background light, medical photos were taken under the same background lighting as possible. In addition, to maintain constant working distance, the border of the picture is framed by an anatomical landmark with about 2 inch background.

### Postoperative care

The first dressing change was performed on postoperative day 4, and thereafter, dressing change was performed every 2 days. During dressing change, the presence of surgical complications such as graft necrosis, graft detachment, or hematoma was evaluated. In case of hematoma, a skin incision was made using a scalpel blade, and the hematoma was removed through saline irrigation, and the dressing was changed again. Easytact® and NPWT were maintained until graft stabilization was achieved. In most cases, NPWT was discontinued at approximately 8–12 days, and dressing change was performed using antibiotic ointment and polyurethane foam. The splint was maintained for up to 2 weeks postoperatively. Skin graft loss rate was measured 14 days after the surgery. The wound photograph was collected and graft loss rate was estimated using ImageJ® (Rasband, W.S., ImageJ, U. S. National Institutes of Health, Bethesda, MA, USA).

## Results

STSG was performed in 41 patients in total, 33 of whom were men and 8 were women (Table 1). Defect location was on the lower extremities in 32 patients, on the upper extremities in 7 patients, and on the pubis and abdomen in 1 patient each. Skin loss rate was measured 14 days after the surgery, and the average skin loss rate was 9.1%, showing a graft take rate of > 90% (Table 2). Also, there were no findings suspicious of immunologic complications caused RMADM.

**Table 1:** Patient demographics

Characteristic	No. of patients
Total no. of patients	41
Age, yr	
Mean	52.9 (17–83)
Sex	
Male	33
Female	8
Cause of skin defect	
Trauma	36
Diabetic foot	2
Release of scar contracture	2
Excision of malignant tumor	1
DM	6
HTN	9
Current smoker	10
ASA class	
I	25
II	15
III	1

*DM* diabetes mellitus, *HTN* hypertension, *ASA* American Society of Anesthesiologists

**Table 2:** Procedural information and outcome

Procedural information and outcome	Value
Defect location	
Lower extremity	32
Upper extremity	7
Others	2
Defect size	
Mean (range)	109.6 cm^2^ (8–450 cm^2^)
Dressing method	
NPWT	36
Tie-over	5
Staged operation	4
Mean interval between ADM and STSG	11
Skin loss rate at 14 postoperative days	9.1%

*NPWT* negative pressure wound therapy, *ADM* acellular dermal matrix, *STSG* split thickness skin graft

## Case report

### Case 1

**Fig. 3:**
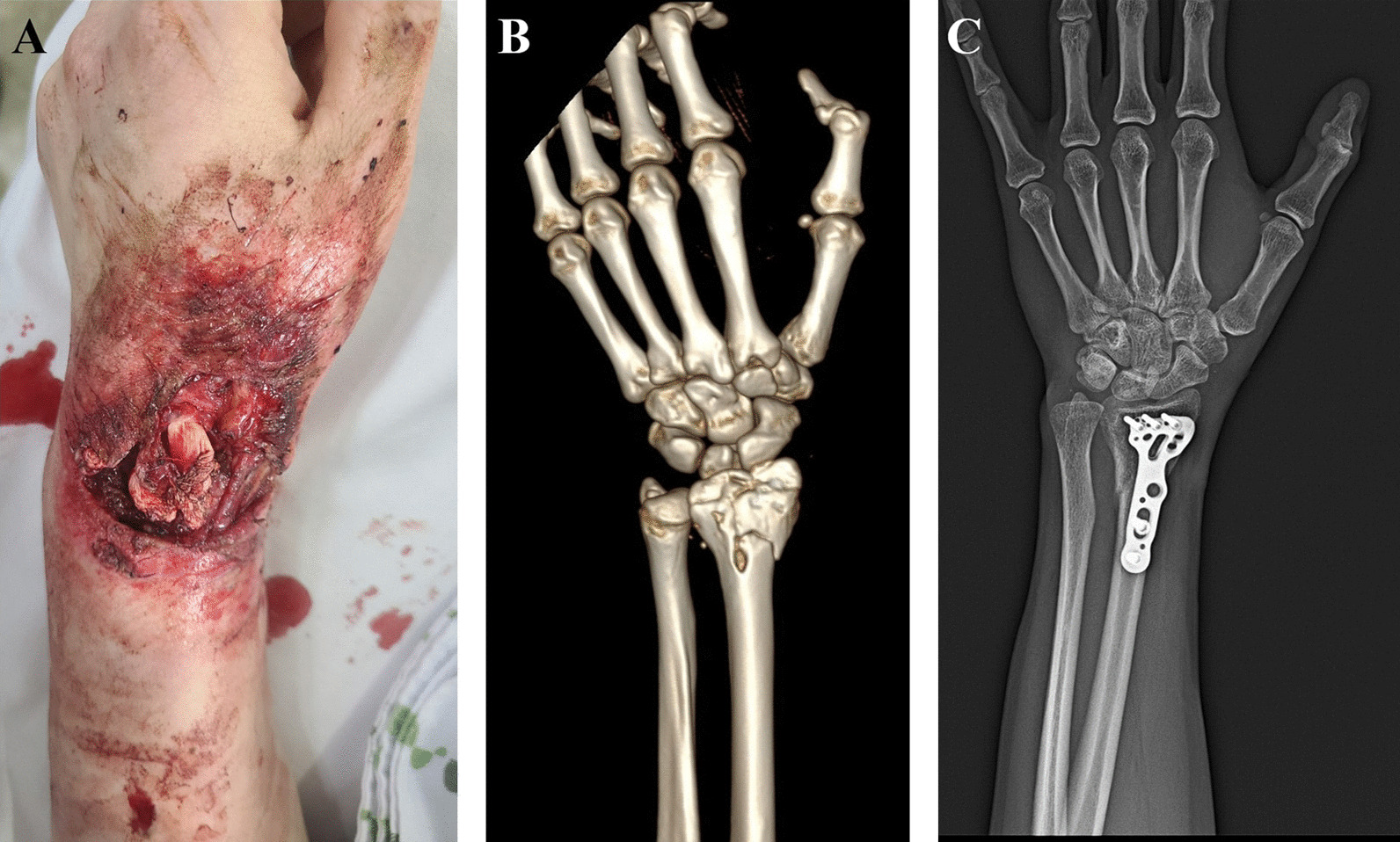
A 41-year-old woman with left wrist wound. **A** Initial wound. **B** CT scan of the fractured bone. **C** X-ray after internal fixation

**Fig. 4:**
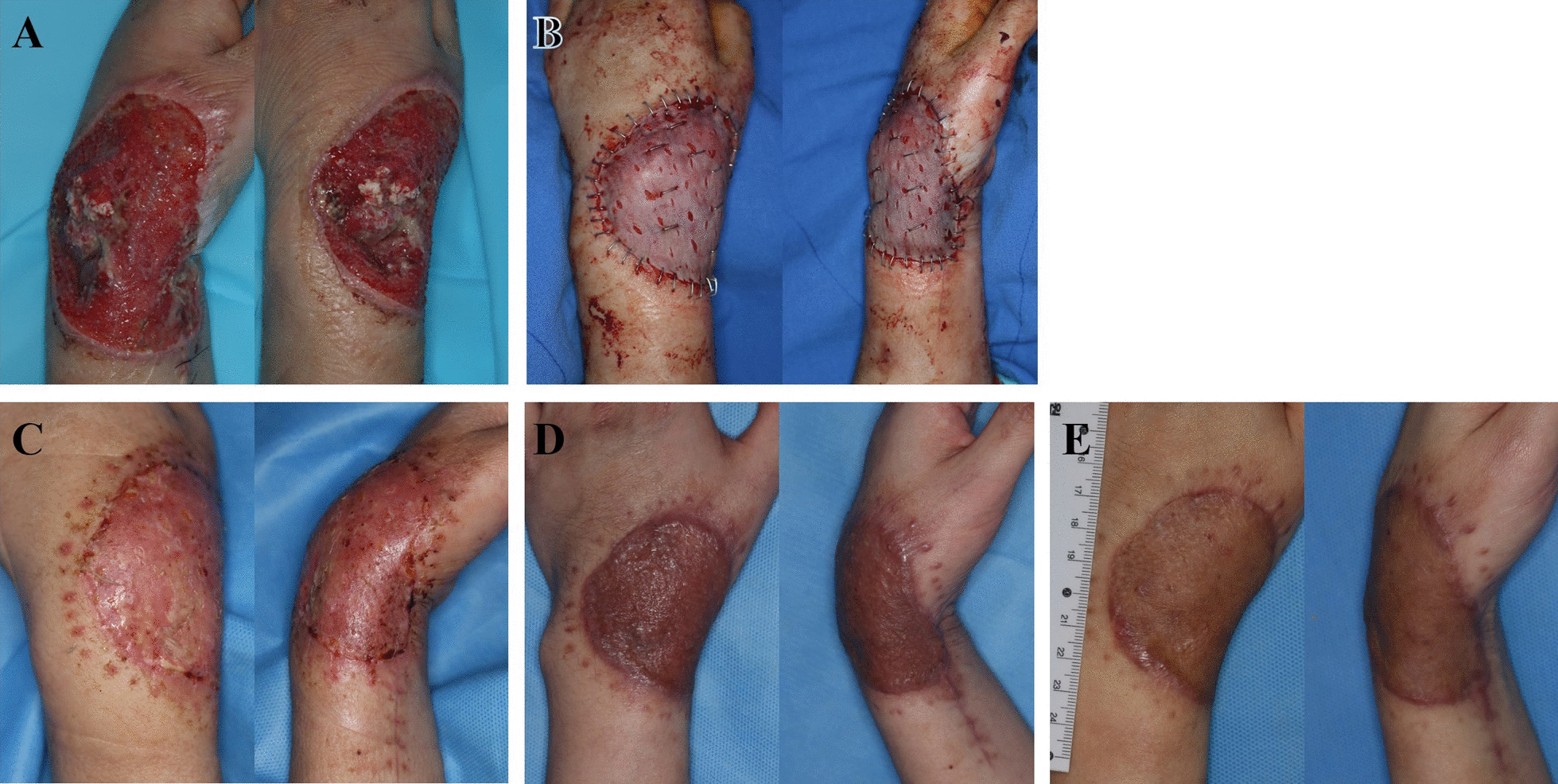
Case 1. **A** Wound before split-thickness skin graft (STSG). **B** Immediate postoperative photo after STSG co graft with RMADM, **C** 10 days postoperatively, **D** 6 weeks postoperatively, and **E** 4 months postoperatively

A 41-year-old woman with a history of major depressive disorder visited our hospital with an open fracture on her left wrist due to a traffic accident. At admission, the wound showed tendon rupture, and computed tomography (CT) showed comminuted fractures of the distal radius and ulna. On the day of admission, debridement was performed by the orthopedic surgeon. Open reduction and internal fixation were performed to the distal radius using a volar-locking plate on the fifth day after trauma (Fig. 3). NPWT was performed for approximately 3 weeks to promote granulation tissue growth. After a long wound preparation period, the patient was finally ready for surgery. Despite tendon exposure, skin grafting was performed using RMADM. The first dressing change was performed on postoperative day 4, and the skin staple was removed on postoperative day 10. She visited the outpatient department at 6 weeks and 4 months after surgery, and skin texture was significantly improved at the last visit. Moreover, in the pinch test, the graft had a soft texture, and there was no limitation in wrist flexion due to graft contracture (Fig. 4).

### Case 2

**Fig. 5:**
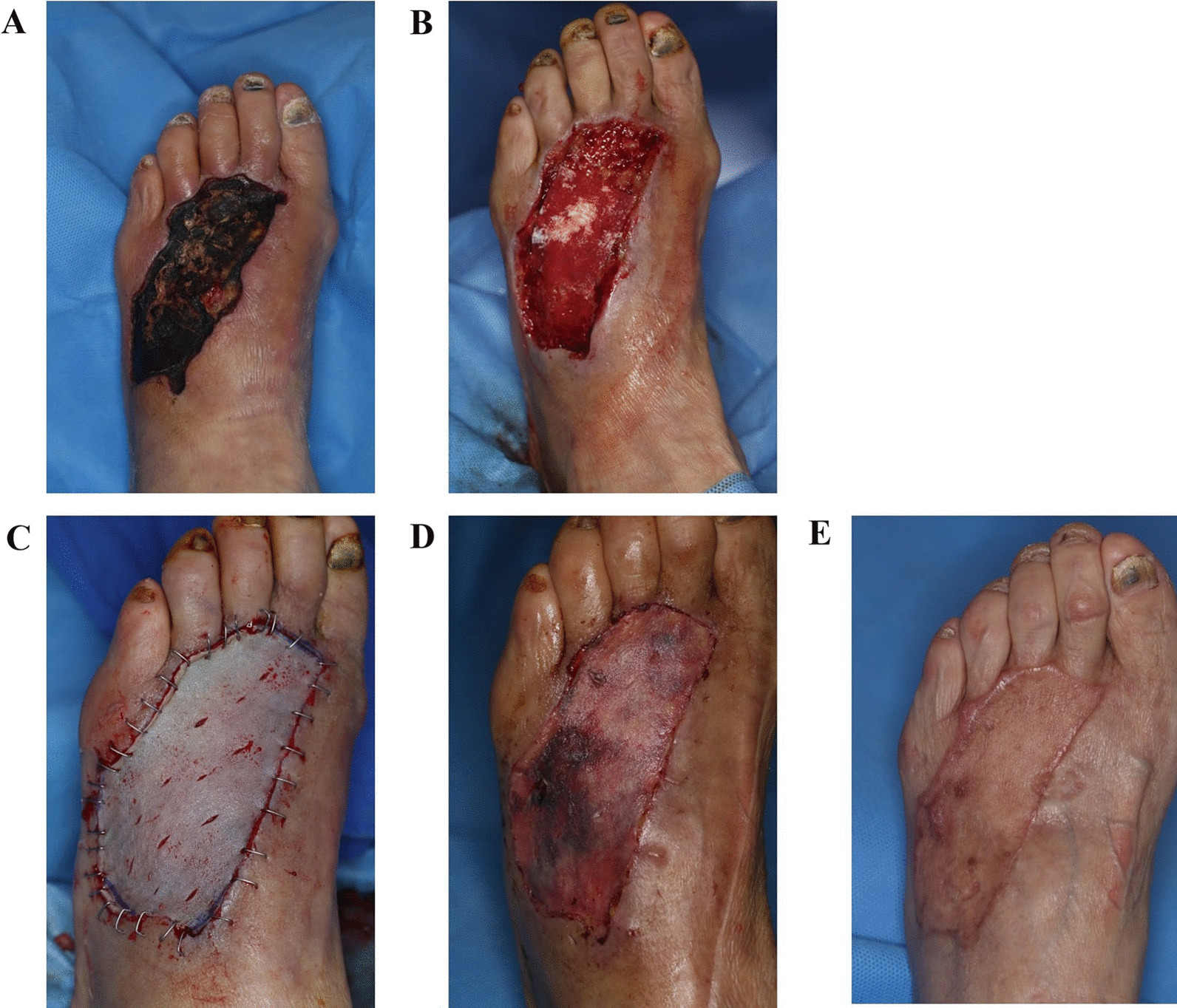
Case 2. **A** Three weeks after trauma and visit to the emergency department. **B** Debridement and RMADM graft. **C** Immediate postoperative photo after delayed split-thickness skin graft, **D** 14 days postoperatively, and **E** 2 months postoperatively

A 78-year-old woman with a history of DM, cerebrovascular accident, and arrhythmia visited the emergency department. Approximately 3 weeks before her visit, a car ran over her forefoot and she visited the local orthopedic surgeon, but her bones were unaffected and only splints were maintained. After the trauma, blistering wounds formed on the skin, and as recommended by traditional medicine, her feet were soaked in water in which alum was dissolved. However, the wound turned black, and she was admitted to the emergency room. On lower extremity CT angiography, no significant abnormalities were observed below the knee artery. Debridement and RMADM graft were first performed under local anesthesia; NPWT was maintained for 1 week and thereafter STSG co graft with RMADM was performed (Additional files 2, 3, 4). During the first dressing change, approximately 10% of graft necrosis was observed. Healing was promoted through secondary intention without additional surgery. Two months after the surgery, a small scar was detected where the graft necrosis had been present previously, but the wound was completely healed and showed excellent skin texture; moreover, there was no limitation in toe motion without graft contracture (Fig. 5).

### Case 3

**Fig. 6:**
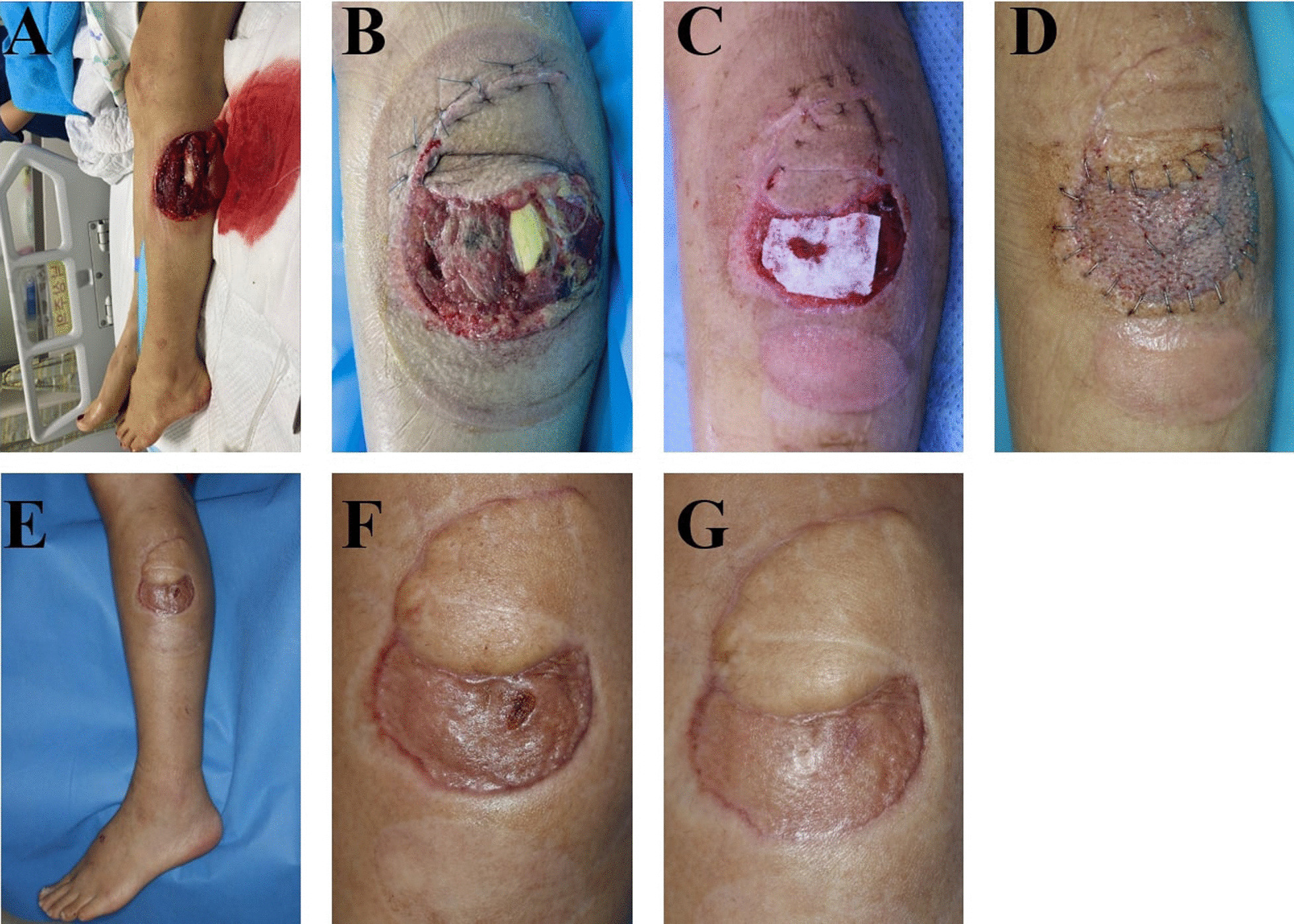
A-54-year-old woman with degloving injury in her left lower leg. **A** Initial wound status. Fibula fracture was present. **B** Image after suture of the degloved tissue. **C** RMADM was applied to cover the tendon. **D** Four days after split-thickness skin grafts (STSG), (**E** and **F**) 1 month after STSG, and **G** 2 months after STSG. Note that a slightly high graft was added in the region of CGDM.

A 41-year-old woman visited our hospital with a degloving injury on her left lower leg due to a traffic accident. Tendon was exposed through ruptured muscle. Orthopaedic surgeons partially repaired degloved flap and tendon could not be covered with the flap. Debridement, RMADM graft and STSG was done simultaneously. First dressing change was performed in day four postoperatively and NPWT was maintained for 8 days to stablize STSG. Two months after the surgery, the tendon was successfully covered with RMADM (Fig. 6).

## Discussion

STSG is a relatively easy and effective method for the reconstruction of a wide range of skin defects. However, it has a disadvantage that it cannot be used when structures such as tendons or bones are exposed. In addition, even if wound healing is successfully achieved through STSG, motion may be restricted due to graft contracture, and a scar with an unfavorable texture may remain.

Antigens and cells that can cause an immune response are removed in the dermal layer in ADM. Further, ADM contains an ECM mainly composed of collagen and elastin. Recently, attempts to use ADM to improve the shortcomings of STSG are increasing. According to Lee et al., the Vancouver scar scale and patient and observer scar assessment scale scores were statistically significantly lower in the case of combined ADM and STSG than in the case of only STSG [8]. Yi et al. confirmed that the pliability score and total score of the Vancouver scar scale were significantly lower in the combined ADM and STSG group [9]. Park et al. conducted a study by assigning patients into a group that received only STSG, a group that received CGDerm^®^ and STSG, and a group that received AlloDerm^®^ and STSG. The scar was objectively evaluated using a Cutometer or a Corneometer, and was not limited to the Vancouver scar scale, which is a subjective evaluation. As a result, better results were obtained both subjectively and objectively in the group in which both ADM and STSG were administered compared with the STSG only group. However, there was no significant difference between the two types of ADM [2].

In addition to these scar improvement effects, ADM has the advantage that it can be used when tendons or bones are exposed. Despite the numerous advantages of STSG, the fact that STSG cannot be performed directly on bones or tendons is a major disadvantage. In particular, in trauma patients with large soft-tissue defects and surrounding tissue belonging to the zone of injury, when STSG cannot be performed, the next option is often a free flap. Free flap is relatively difficult to perform, is time consuming, and is associated with the possibility of failure. Further, its donor site morbidity is higher than that of STSG and it has the disadvantage of having a bulky contour.

However, using ADM, STSG can be successfully performed even when tendons or bones are exposed. As confirmed in the case reports above, when vascularized tissue is present in the periphery, the tendon can be successfully covered with single-stage surgery, and the STSG can take well to the surrounding tissues.

Previously also the co-graft of ADM and STSG has been done frequently, and the literature on this has been easily found and mentioned above. However, RMADM is a completely new type of ADM and has several advantages over existing ADM or dermal substitutes. Existing dermal substitutes such as Insuregraf^®^ and Matriderm^®^ are manufactured from porcine and bovie dermis. On the other hand, RMADM is more biocompatible because it is fabricated through the human dermis. Also, dermal substitutes such as Matriderm^®^ or Insuregraf^®^ are relatively easy to use with STSG in single-stage surgery, but are not physiologically more similar compared to ADM, and whether the collagen material remains engrafted for a long time has not been histologically studied.

RMADM does not increase the failure rate of STSG according to this study. Although there is controversy, the graft failure rate increases in the case of STSG co graft with ADM especially in single-stage surgery. In previous studies, ADMs such as AlloDerm^®^ or CGDerm^®^ were widely used. These ADM types can be effective when taken well, but are often difficult to take. In particular, when STSG and ADM are performed together in single-stage surgery, ADM can interfere with plasma imbibition, thus causing further graft loss. In particular, unmeshed STSG is more difficult to take [10–14]. However, RMADM is a sheet type manufactured by freeze-drying and has good permeability with network structure unlike the other ADM such as AlloDerm^®^. Therefore, complications such as hematoma and seroma are low, and plasma imbibition is not blocked, so it does not affect the graft failure rate.

In addition, RMADM is more economical than traditional ADM. In the case of a large size ADM, there is a limitation in production because a wide skin donor is required. For example, in case of full coverage of the implant with ADM in implant based breast reconstruction, a wide ADM of 400 cm^2^ or more may be required depending on the size of the implant. ADM is made through skin donation, and it is not easy to obtain such a wide size and uniform thickness, which may limit production and lead to an increase in price. However, RMADM is manufactured by freeze-drying and pulverizing the existing ADM. Therefore, it is possible to assemble small pieces of ADM by-product produced during the manufacturing process. It can be manufactured at a low price because it can be manufactured without affecting the width or thickness of the donor’s skin.

Even when performing actual surgery, RMADM has several distinct advantages. First you can adjust the thickness. The RMADM used in this study has a thickness of about 0.2 mm. If the wound requires the use of thicker ADM, the thickness can be easily adjusted by layering multiple layers of RMADM. The size is also easily adjustable. Existing ADM was not easy to cover a wider wound than the size of ADM. However, as RMADM is a sheet type, it can be easily shredded and deformed to fit the wound size. As an example, a photo of RMADM applied to the wound before STSG after mastectomy and video of surgical procesure is attached (Additional files 1, 2, 3, 4). If you look closely, you can see that a large square RMADM was cut into several pieces and applied to fit the size of the round (Additional file 1). Lastly, unlike ADM, RMADM does not require a fixation process. It can be applied easily by attaching to the wound and hydrating it through saline irrigation. This can shorten the operation time.

**Fig. 7:**
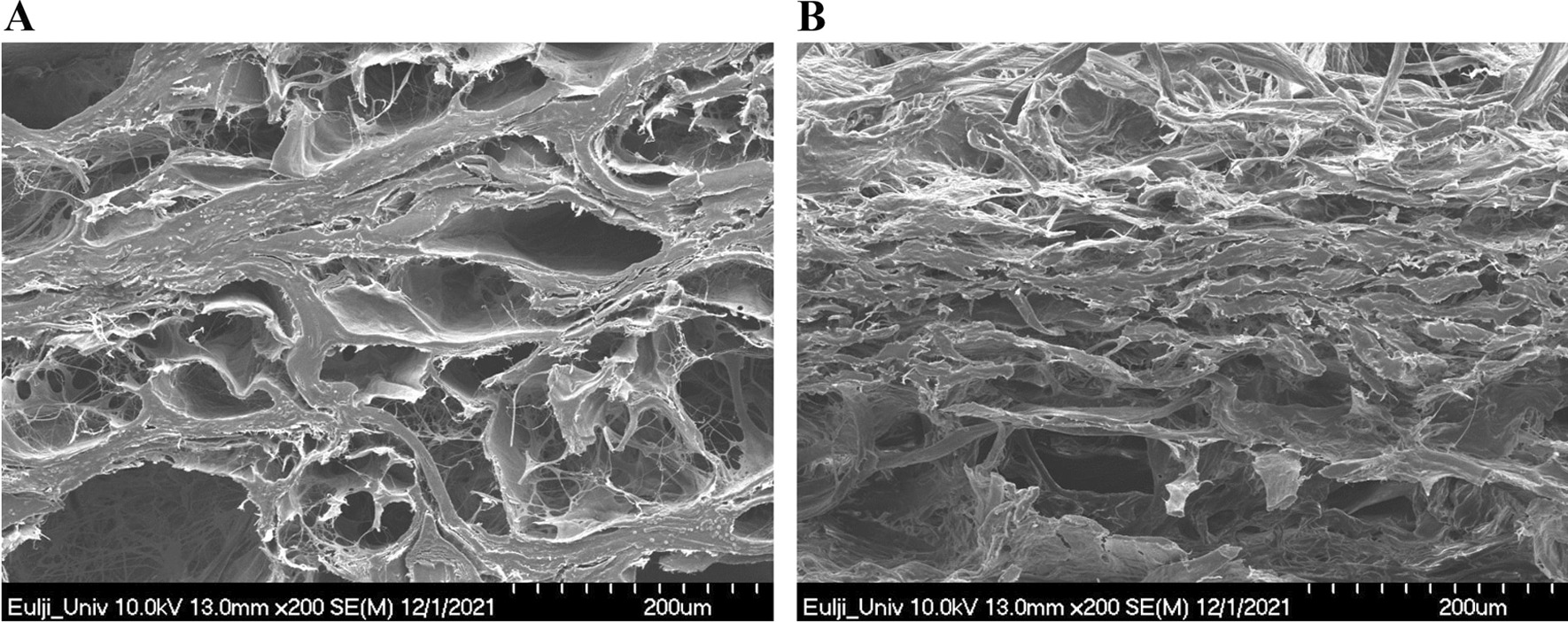
Microstructure of the CGDerm matrix^®^ (CGDM) compared with the structure on electron microscopy. **A** CGDerm^®^. **B** CGDM. The extracellular matrix-like network structure was preserved in the CGDM

RMADM, a novel ADM, is manufactured by freeze-drying ADM, pulverizing it into a fiber type, and reprocessing it into a sheet form. Because it is pulverized into fiber type rather than particle type, RMADM can be reprocessed into a sheet form, and therefore, it has a shape closer to the structure of ECM. When compared using electron microscopy, the structure of RMADM shows a network similar to that of the ECM in CGDerm^®^ (Fig. 7). RMADM is a micronized ADM that is relatively easy to be taken in wounds and does not interfere with plasma imbibition compared with conventional ADMs such as CGDerm^®^ or Alloderm^®^. Nevertheless, similar to other ADMs, RMADM contains collagen, elastin, and proteoglycan, and it is therefore more physiologically similar than other dermal substitutes such as Matriderm^®^ and Insuregraf^®^, which are artificially processed from bovine dermis.

**Fig. 8:**
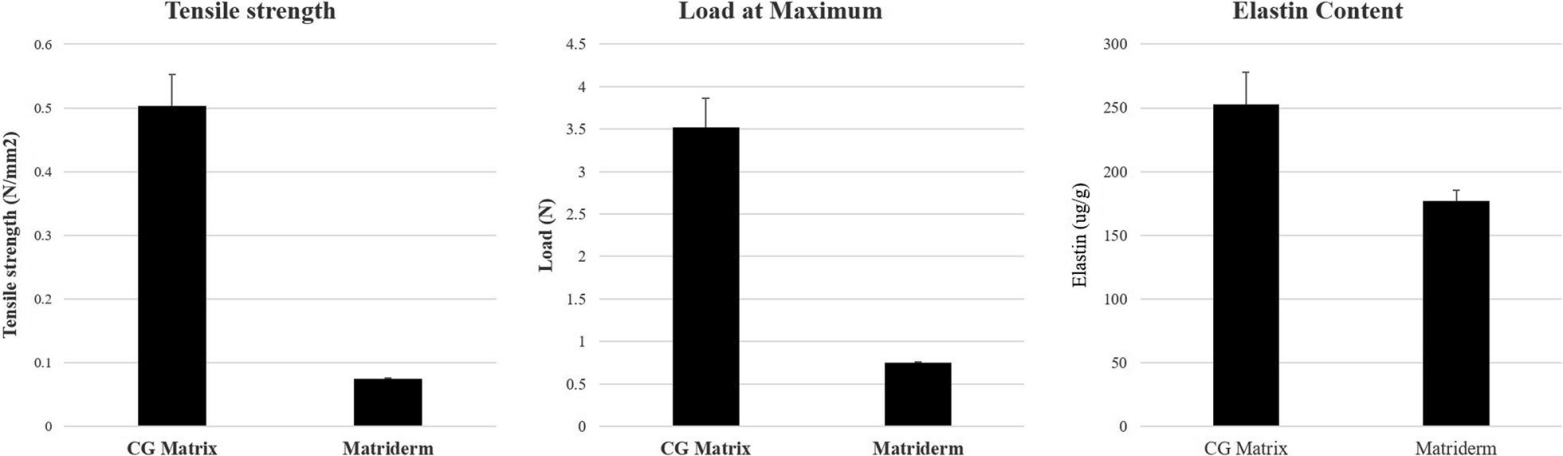
**A** Tensile strength of the CGDerm matrix^®^ (CGDM) compared with Matriderm. **B** Maximum load before breakage. **C** Elastin content

In addition, RMADM has a higher elastic content than Matriderm^®^ of the same weight. Examination of elastin content using ELISA revealed that RMADM contains 252 μg of elastin per 1 g, which is 43% higher than that of Matriderm^®^, which contains 176 μg of elastic per 1 g. Elastin is a protein that plays an important role in maintaining skin elasticity and is expected to play a major role in preventing graft contracture (Fig. 8).

RMADM has a stronger tensile strength than Matriderm^®^ (Fig. 8) probably because it forms a network structure resembling that of ECM. One of the main areas for using combined ADM and STSG is in the joint site where graft contracture is fatal. If both RMADM and STSG are applied to the joint, the strong tensile strength of RMADM will provide sufficient strength to the wound and reduce scar widening.

RMADM does not interfere with plasma imbibition, even when used together with STSG in single-stage surgery. In addition, it has a network structure similar to that of ECM and has high elastin content and high tensile strength. Therefore, it can reduce graft contracture and increase wound strength. Additionally, it has the characteristics of an ideal ADM for skin graft use.

The limitation of this study is neglect of scar assessment after wound repair. However, this study was intended to be meaningful in confirming whether novel RMADM with these distinct advantages can be applied to clinical practice for the first time. The authors of this paper consider RMADM to be a game-changing novel material for ADM used with STSG. Further study comparing the difference in scar where RMADM is co-grafted and only STSG is applied will be needed.

## Conclusion

RMADM is a sheet-type ADM, and it is easier to co-graft compared with previous ADMs. Further, it does not interfere with plasma imbibition, even when used together with STSG in single-stage surgery. In addition, it has an ECM network structure that is similar to that of existing ADMs and has a high elastin content and high tensile strength. It can therefore reduce graft contracture and increase wound strength. Finally, it has the characteristics of an ideal ADM for skin graft use.

## Supplementary information

**Additional file 1: Fig. S1.** (Above) immediate postoperative photo of mastectomy. (Below) RMADM is applied cut in to the shape of the wound.

**Additional file 2: Video S1. **Sharp debridement of left foot wound.

**Additional file 3: Video S2. **Applying RMADM. Note that it does not need fixation and can be cut in to the shape of the wound.

**Additional file 4: Video S3. **STSG over RMADM in a single-stage.

## Data Availability

The datasets generated and/or analysed during the current study are not publicly available due to patient privacy protection but are available from the corresponding author on reasonable request.

## Abbreviations

ADM: Acellular dermal matrix
ASA: American Society of Anesthesiology
CGDM: CGDerm matrix^®^
CT: Computed tomography
DM: Diabetes mellitus
ECM: Extracellular matrix
NPWT: Negative pressure wound therapy
RMADM: Reprocessed micronized ADM
STSG: Split thickness skin graft
