# Supplementary figures and images for: Efficacy of split-thickness skin graft combined with novel sheet-type reprocessed micronized acellular dermal matrix
Source: BMC Surg. 2022 Oct 11;22:358. doi: 10.1186/s12893-022-01801-x (PMC9555098; doi:10.1186/s12893-022-01801-x)

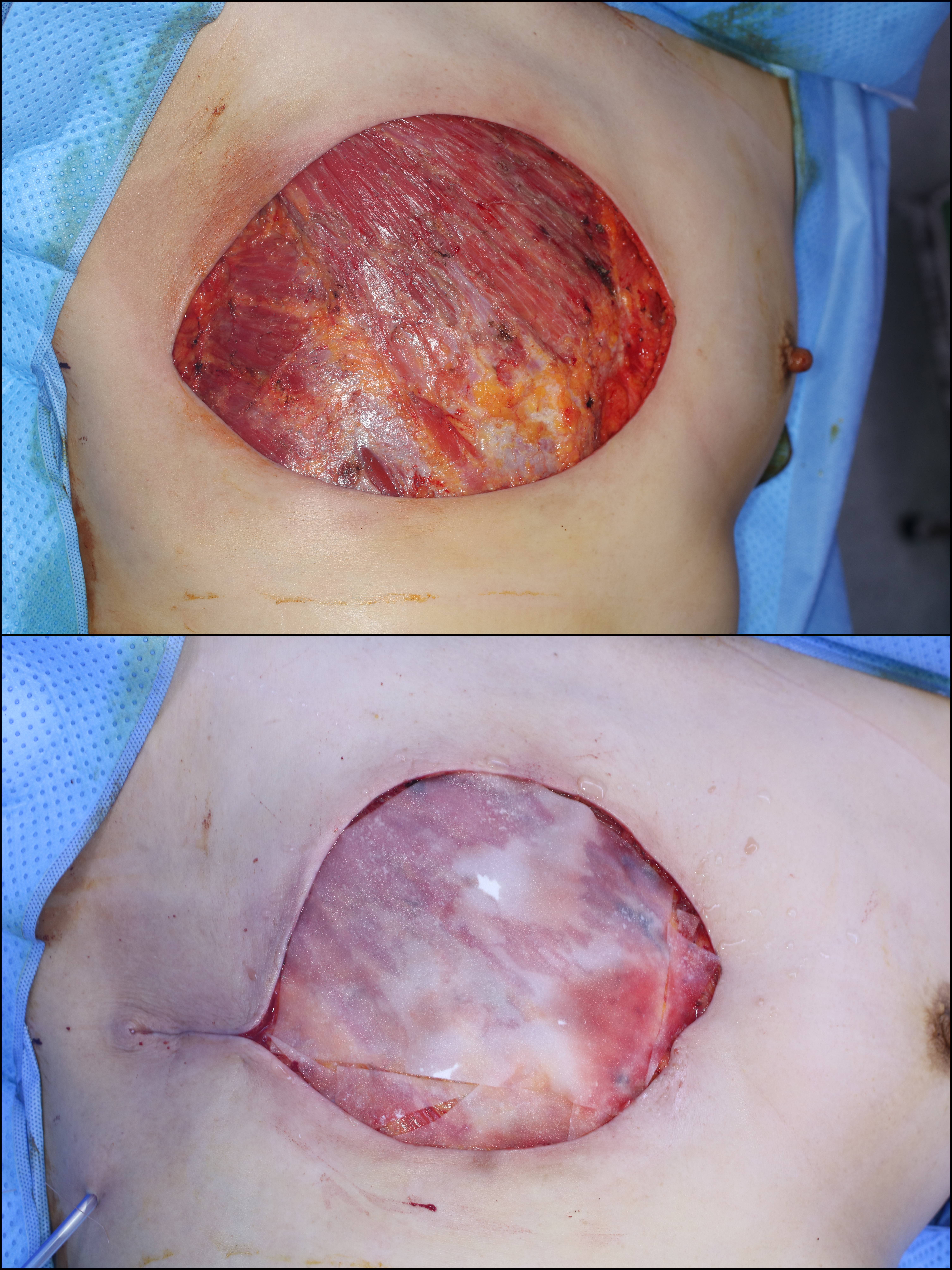

Supplement: Supplementary file 1 — Additional file 1: Fig. S1. (Above) immediate postoperative photo of mastectomy. (Below) RMADM is applied cut in to the shape of the wound. [file 12893_2022_1801_MOESM1_ESM.jpg]
